# Barriers to cervical cancer prevention in a safety net clinic: gaps in HPV vaccine provider recommendation and series completion among Ob/Gyn patients

**DOI:** 10.3389/fonc.2024.1359160

**Published:** 2024-03-28

**Authors:** Lindsey A. Finch, Morgan S. Levy, Amanda Thiele, Patricia Jeudin, Marilyn Huang

**Affiliations:** ^1^ Department of Obstetrics, Gynecology, and Reproductive Sciences, Jackson Memorial Hospital, Miami, FL, United States; ^2^ Department of Medical Education, University of Miami Miller School of Medicine, Miami, FL, United States; ^3^ Division of Gynecologic Oncology, Sylvester Comprehensive Cancer Center/University of Miami Miller School of Medicine, Miami, FL, United States

**Keywords:** human papilloma virus, cervical cancer, vaccination, patient education, healthcare disparities

## Abstract

**Objective:**

The primary objective of this study was to evaluate patients’ knowledge regarding HPV vaccination and vaccine uptake in a diverse patient population. The secondary objective was to evaluate factors influencing the decision to vaccinate, potential barriers to vaccination, and to assess whether HPV vaccines were offered to or discussed with eligible patients in a safety net Obstetrics and Gynecology (Ob/Gyn) clinic.

**Methods:**

A 28-item survey was developed using Likert scale survey questions to assess patient agreement with statements regarding HPV and the vaccine. The surveys were administered to patients in the Ob/Gyn outpatient clinics from May 2021 through September 2022. Additionally, pharmacy data were reviewed and chart review was performed as a quality improvement initiative to assess the impact of expanded HPV vaccine eligibility to patients with private insurance on vaccine uptake. Descriptive statistics were performed.

**Results:**

304 patients completed surveys from May 2021 through September 2022. The median age of respondents was 32 (range 18-80). 16 (5%) were Non-Hispanic White, 124 (41%) were Hispanic White, 58 (19%) were Non-Hispanic Black, 6 (2%) were Hispanic Black, 29 (9.5%) were Haitian, 44 (14%) were Hispanic Other, 7 (2%) were Non-Hispanic Other, 20 (6.6%) did not respond. 45 (14%) patients were uninsured. Many patients (62%) reported that a physician had never discussed HPV vaccination with them. Seventy nine percent of patients reported they had never received the HPV vaccine, and 69% of patients reported that lack of a medical provider recommendation was a major barrier. Among patients to whom HPV vaccination had been recommended, 57% reported that the vaccine was not available the same day in clinic.

**Conclusion:**

Our study demonstrated that many patients never had a provider discuss HPV vaccination with them and never received the HPV vaccine. Additionally, amongst those who did initiate HPV vaccination, completion of the series remains a key barrier. Ensuring that providers discuss HPV vaccination and that patients receive HPV vaccines, along with expanding access to and convenience of HPV vaccination are critical aspects of preventing cervical cancer.

## 1 Introduction

The human papillomavirus (HPV) is the leading cause of cervical cancer (1, 2). In the United States (U.S.), a virus-like particle-based vaccine has been available since 2006, with the 9-valent vaccine available since 2016 that provides protection against 9 strains of high-risk HPV (3, 4). Increasing uptake of the HPV vaccine is an important aspect of the World Health Organization’s (WHO) goal of eradicating cervical cancer (5). The WHO has set a target of vaccinating 90% of women by age 15 (6). The U.S. Department of Health and Human Services has similarly set a target of 80% of adolescents receiving HPV vaccines as part of its Healthy People 2030 initiative (7). The Advisory Committee on Immunization Practices and the American College of Obstetricians and Gynecologists (ACOG) recommend HPV vaccination for all men and women between the ages of 9-26, and with shared decision-making for patients ages 27-45 (8).

However, despite the wide availability of HPV vaccination in the U.S., vaccination rates are low, with only 27% of men and 53.6% of women between the ages of 18-26 reporting vaccination (9). Furthermore, there are significant racial and ethnic disparities in knowledge of and access to the vaccine (10). Associations have been noted between HPV knowledge and factors such as race, ethnicity, nation of origin, level of education, and primary language (11, 12). Racial disparities are significant barriers in women’s healthcare, likely contributing to these differences (13). Both incidence and death rates from cervical cancer reflect racial and ethnic disparities (14). From 2016-2020, the U.S. incidence rate among Hispanic and Non-Hispanic Black (NHB) patients was 9.3 and 8.5 respectively, compared to 7.1 for non-Hispanic White (NHW) patients (7). From 2016-2020, the death rates for NHB and Hispanic patients were 3.3 and 2.5 respectively, while the death rate for NHW patients was 2.0 (7). Hispanic women are 1.26 times more likely to die of cervical cancer than NHW women (15).

In addition to primary prevention, HPV vaccination may also be utilized in the adjuvant setting for the management of cervical dysplasia. In 2023, ACOG expanded recommendations for consideration of HPV vaccination for patients undergoing treatment of CIN 2 (16). Recent studies have demonstrated that the majority of patients who developed lower genital tract dysplasia following hysterectomy for HPV-related disease had HPV subtypes that would be covered by the HPV vaccine, supporting further investigation of adjuvant vaccination (17). The management of HPV-associated cervical dysplasia, including increased clinical surveillance, biopsies, and excisional procedures such as loop electrosurgical excision procedure (LEEP) and cervical conization, contributes to heightened patient distress and anxiety (18). Excisional procedures have also been associated with adverse pregnancy outcomes including cervical insufficiency, preterm labor, and preterm delivery (19). Counseling patients about HPV prevention is unique because HPV is not only a sexually transmitted infection, but more importantly, causes a significant burden of disease through precancer and cancer in both men and women (10). Persistence of HPV infection is one of the most significant risk factors for developing HPV-related dysplasia and malignancy (20). Assessing patients’ understanding of HPV and HPV vaccination will help providers better counsel patients to accept vaccination for cancer prevention.

The primary objective of this study was to evaluate patients’ knowledge of HPV vaccination and vaccine uptake in a diverse patient population. The secondary objective was to evaluate factors influencing the decision to vaccinate, potential barriers to vaccination, and to assess whether HPV vaccines were offered or discussed during clinic visits for eligible patients in a safety net Ob/Gyn clinic.

## 2 Materials and methods

Institutional review board approval was obtained (IRB #00058353). A 28-item survey was developed using Likert scale survey questions to assess patient agreement with statements regarding HPV and the HPV vaccine from May 2021 through September 2022. The anonymous surveys were then given to patients in the Ob/Gyn outpatient clinics while waiting for their appointments. Patients had the option of not participating by not completing the survey. Patients had to be 18 years or older, be able to read English, Spanish, or Haitian Creole and be able to self-complete the survey. Demographic data was self-reported by survey participants, and racial and ethnic categories were chosen by investigators in order to capture the diverse racial and ethnic composition of our patient population (21). Study data was collected and managed using REDCap electronic data capture tools hosted at the University of Miami Miller School of Medicine. REDCap (Research Electronic Data Capture) is a secure, web-based software platform designed to support data capture for research studies by providing audit trails for detecting data manipulation, automated export procedures and systems for integration with external sources (22, 23).

At the beginning of the study period, only patients with county funding were able to receive HPV vaccination in the Ob/Gyn clinics. However, following significant engagement with hospital leadership, starting in September 2021, HPV vaccination was expanded to patients with commercial insurance. Thus, the timeframe, Aug 2020 to Aug 2021 was determined as standard care for patients with county insurance, while Sept 2021 to Sept 2022 as expanded access to patients having commercial insurance in addition to those with county insurance. Patients without insurance were not able to receive HPV vaccination during either time period.

Separate from the anonymous surveys as a quality improvement initiative, chart review and analysis of pharmacy data were used to assess the completion of the HPV vaccine series from August 2020 - September 2022 within the clinic. The time period of study was chosen to assess vaccination uptake in the year prior to and following expanded access to vaccination for patients with insurance. Descriptive statistical analysis was performed to evaluate frequency, range, mean, and median values.

## 3 Results

### 3.1 Demographics

A total of 304 patients presenting for an Ob/Gyn clinic visit completed surveys. Of the respondents, 124 (41%) self-identified as Hispanic White (HW), 16 (5%) as NHW, 58 (19%) as Non-Hispanic Black (NHB), 6 (2%) as Hispanic Black (HB), 29 (9.5%) as Haitian, 44 (14%) as Hispanic Other (HO), 7 (2%) as Non-Hispanic Other (NHO) and 20 (6.6%) as No Response (NR) (**Table 1**). With respect to insurance status, 112 (34%) patients had Medicaid, 45 (14%) patients were uninsured, 38 (12%) had hospital safety net funding, 39 (12%) had Medicare and 29 (9%) had private insurance. The median age of respondents was 32 (range 18-80).

**Table 1:** Demographics.

	Participants (n=304)
Race/Ethnicity Hispanic White Non-Hispanic White Non-Hispanic Black Hispanic Black Haitian Hispanic Other Non-Hispanic Other No response	124 (41%) 16 (5%) 58 (19%) 6 (2%) 29 (9.5%) 44 (14%) 7 (2%) 20 (6.6%)
Insurance status Medicaid Uninsured Hospital Safety Net Funding Medicare Private Insurance Other No response	112 (34%) 45 (14%) 38 (12%) 39 (12%) 29 (9%) 16 (5%) 25 (8%)
Age Mean Median Range	34 32 18-80

### 3.2 HPV knowledge

Only 45% (n=138) of patients knew that HPV causes cancer (**Table 2**). While 213 (70%) patients identified at least one correct route of transmission including vaginal, anal, or oral sex and associated skin-to-skin contact, 78 (26%) patients reported that they did not know the route of HPV transmission. Over half (n=183, 60%) of patients identified at least one health consequence of HPV infection, while 106 (35%) patients reported that they did not know the health consequences of HPV infection.

**Table 2:** HPV Knowledge by Race and Ethnicity.

	All (n,%)	Hispanic White	Non-Hispanic White	Black (Hispanic)	Black (Non-Hispanic)	Haitian	Other (Hispanic)	Other (Non-Hispanic)	No Response
**Total**	304	124 (41%)	16 (5%)	6 (2%)	58 (19%)	29 (9.5%)	44 (14%)	7 (2%)	20 (6.6%)
**Knew HPV causes cancer**	138 (45%)	61 (37%)	9 (56%)	4 (67%)	23 (40%)	11 (38%)	21 (47%)	2 (29%)	7 (35%)
**Correctly identified at least one route of HPV transmission**	213 (70%)	100 (81%)	12 (75%)	6 (100%)	29 (50%)	11 (38%)	38 (86%)	6 (86%)	11 (55%)
**Not know health consequences of HPV infection**	106 (35%)	35 (28%)	5 (31%)	1 (17%)	29 (50%)	13 (45%)	14 (32%)	2 (29%)	7 (35%)
**Knew oldest age of vaccine eligibility**	22 (7%)	7 (5.6%)	1 (6%)	0 (0%)	3 (5%)	5 (17%)	5 (11%)	0 (0%)	1 (5%)
**Knew correct number of doses**	52 (17%)	18 (15%)	3 (19%)	2 (33%)	12 (21%)	8 (28%)	6 (14%)	3 (43%)	0 (0%)
**Knew men were eligible for the vaccine**	113 (37%)	49 (40%)	7 (44%)	4 (67%)	16 (28%)	9 (31%)	20 (45%)	5 (71%)	3 (15%)

Only 22 (7%) patients knew that HPV vaccination eligibility had been extended up to the age of 45. The majority (83%) of patients did not know how many doses of HPV vaccination are required. Few patients (n=113 37%) were aware that men were eligible for HPV vaccination.

### 3.3 HPV counseling, uptake, and vaccine availability

Many patients had never discussed HPV (n=113, 37%) or the HPV vaccine (n=189, 62%) with a healthcare provider (**Table 3**). Additionally, more than half (n=211, 69%) of patients reported that a healthcare provider had never recommended HPV vaccination to them.

**Table 3:** HPV Vaccine Counseling and Uptake.

	All (n,%)	Hispanic White	Non-Hispanic White	Black (Hispanic)	Black (Non-Hispanic)	Haitian	Other (Hispanic)	Other (Non-Hispanic)	No Response
**Total**	304	124 (41%)	16 (5%)	6 (2%)	58 (19%)	29 (9.5%)	44 (14%)	7 (2%)	20 (6.6%)
**Healthcare provider never discussed HPV infection**	113 (37%)	40 (32%)	8 (50%)	0 (0%)	26 (45%)	16 (55%)	14 (32%)	1 (14%)	8 (40%)
**Healthcare provider never discussed HPV vaccine**	189 (62%)	76 (61%)	14 (88%)	3 (50%)	32 (55%)	20 (69%)	25 (57%)	5 (71%)	14 (70%)
**Healthcare provider had never recommended HPV vaccine**	211 (69%)	90 (73%)	14 (88%)	4 (67%)	39 (67%)	15 (52%)	30 (68%)	4 (57%)	15 (75%)
**Did not receive the vaccine**	241 (79%)	100 (81%)	14 (88%)	4 (67%)	46 (79%)	20 (69%)	39 (89%)	3 (43%)	15 (75%)

More than half (n=173, 57%) of all patients reported that they did not believe that the HPV vaccine was available to be given the same day in the clinic, including both those who had and who had not been offered the vaccine. A majority (n=241, 79%) of patients were unvaccinated. Among patients who were not vaccinated reasons cited included not being offered vaccination (n=86, 28%), not being aware that they were eligible for vaccination (n=86, 28%), concerns about toxic ingredients in the vaccine (n=48, 16%), not believing in vaccinations (n=21, 7%), not being able to afford vaccination (n=43, 14%), and perceived inconvenience of receiving the vaccine (n=24, 8%).

### 3.4 Vaccine administration

HPV vaccination was not available to patients with commercial insurance in our safety net clinic prior to September 2021. From September 2021-September 2022, the number of patients receiving HPV vaccination increased compared to the 1 year prior to expansion of eligibility.

## 4 Discussion

Uptake and provider recommendation of HPV vaccination remains low among Ob/Gyn patients at our safety net hospital. The patterns seen in our population mirror the U.S. as a whole, where despite education and outreach efforts, vaccination rates remain below goal (9). HPV-related cancer incidence reflects this suboptimal vaccination rate, with an estimated 47,199 new cases of HPV-associated cancers annually from 2015-2019 (24). A study from 2021 found that only 47.7% NHW women, 30.9% of Hispanic women, 38.1% of Black women, and 25.9% of Asian women aged 18 to 26 years had received vaccination (25). HPV vaccination also differs by nativity, with 27.4% of adults aged 18 to 26 years born in the U.S. reporting vaccination compared to 14.3% not born in the U.S (25).

Prevention of HPV-related cancers has been cited as a priority of both international and U.S. health policy organizations (5–7)., In Australia, where HPV vaccination rates are high and supported through a national vaccination program, the HPV-associated disease burden has been substantially reduced – and is even projected to reach fewer than 4 new cases of cervical cancer per 100,000 by 2028 (26, 27).As follows, directed efforts including broader availability of HPV vaccination in clinical sites serving diverse populations are essential to increasing uptake. Studies have found that many patients receive the majority of their primary care with Ob/Gyn providers, underscoring the importance of ensuring broad availability of and counselling regarding HPV vaccination in these settings (28). The success and barriers faced in implementing vaccination in our clinic highlight considerations for other sites that wish to replicate this model.

### 4.1 Knowledge

HPV knowledge is fundamental to increasing vaccine uptake (29–33). However, HPV and HPV vaccine knowledge in the U.S. remains low, with studies showing that only 68% of adults report having heard of HPV and the vaccine (34, 35). There are significant disparities in knowledge of and access to the vaccine (10). Associations have been noted between HPV knowledge and factors such as race, ethnicity, national origin, and primary language (11). There are multiple barriers to patient knowledge that have been identified in the literature, including language, cost, lack of information, and fear or mistrust of the healthcare system (36–38). While informational interventions can have a positive impact on vaccine uptake (29, 39, 40), educational interventions alone are likely insufficient to significantly improve vaccination rates (29, 41).

Consistent with prior literature, our study at a diverse safety net clinic found that most patients did not know basic information about the HPV vaccine, had not received HPV vaccination and that vaccination had never been discussed with them. High levels of health literacy have been linked to cervical cancer screening behaviors (42). Studies have also shown that health literacy is a stronger predictor of knowledge of risk factors for cervical dysplasia than race and ethnicity (43). Furthermore, increased information has a positive impact on vaccine uptake (39, 40, 44). Understanding the risk of HPV infection and benefits of vaccination has been associated with intention to vaccinate (45, 46).

Most patients in our study did not know that HPV infection could lead to cervical cancer, with a lower percentage of NHO, NHB, HW and Haitian patients, but a higher percentage of NHW, HB and HO patients reporting this knowledge. While this reflects findings of other studies that have demonstrated that NHB patients were less likely to report knowledge of the association between HPV and cervical cancer, it differs from findings among Hispanic patients (47). In contrast to other studies, we found proportionally higher reported knowledge of the association between HPV and cervical cancer among patients identifying as Hispanic other than HW patients (47). This discrepancy possibly reflects the unique demographic composition of Miami Dade County, which is majority Hispanic. Most patients in our study did not know basic information about the HPV vaccine such as ages of eligibility, number of doses, and whether men could receive the vaccine. Given the association between HPV and vaccine knowledge and uptake, it is imperative for healthcare providers to continue to strengthen targeted education efforts for patients and their communities.

### 4.2 Vaccination status

Most patients in our study had not received the HPV vaccine. NHO, HB and Haitian patients had the highest level of vaccine uptake. These results were not consistent with prior studies that have shown that misconceptions regarding HPV and vaccination are extremely common among immigrants from Haiti in the U.S (48). This discrepancy could reflect a higher level of Haitian and Haitian Creole-speaking providers in our population, but conclusions are limited by small sample size. It is important to increase education and outreach efforts, as studies have demonstrated that knowledge is a positive predictor of HPV vaccination (35).

### 4.3 Vaccination counselling

While many patients had discussed HPV with a healthcare provider, most had not discussed HPV vaccination or had the HPV vaccine recommended to them. This result was consistent across racial groups, with the greatest number of Hispanic patients reporting having discussed HPV and HPV vaccination. Other studies have found that many Hispanic and Haitian patients have not received vaccination recommendations from providers, and that patients with limited English proficiency were most affected (49, 50). The discrepancy in our data from these national studies likely reflects the unique patient and provider population of Miami Dade County, where the majority of the population is Hispanic. This is consistent with previous studies that have found significantly better outcomes among Hispanic patients with cervical cancer in Miami Dade County compared to nationally (51). Patient understanding and satisfaction increase in environments with culturally and linguistically concordant care (52, 53). Our data support creating clinical environments where providers and support staff have the linguistic ability and cultural competency to properly counsel patients.

Multiple studies have shown that a strong recommendation from a healthcare provider has a positive impact on vaccine uptake (50, 54–56). Furthermore, patients who report receiving HPV information from a physician have been found to have higher knowledge scores (35). In our study among those patients who were not vaccinated, lack of strong recommendation from a healthcare provider and lack of awareness of eligibility were cited as significant barriers. Additionally, previous work has shown major deficits in knowledge of the HPV vaccine among students in the health professions and healthcare providers (57). Even among patients in gynecologic oncology clinics, providers are less likely to offer vaccination to patients without HPV-related dysplasia (58). It is critical to ensure that healthcare providers are knowledgeable about HPV vaccination, up to date on current practice guidelines, and trained in counselling patients given the recent increase in vaccine hesitancy (59).

### 4.4 Vaccine administration

The quality improvement initiative performed in connection with our study noted an increase in HPV vaccine administration following expansion of eligibility to patients with insurance. Although many of our patients reported that they did not believe that the HPV vaccine was available for same-day clinic administration, uptake increased with expanded eligibility. The convenience of administration is a known barrier to vaccine acceptance, administration, and series completion (60, 61). Interventions such as designated provider champions, clinical screening, financial assistance and elimination of barriers such a pregnancy testing have been shown to improve vaccine uptake (62). A limited number of patients completed the recommended 3-dose vaccine series, which is consistent with national trends (63–65). The percentage of women age 18 to 26 who received the recommended number of doses has been increasing, from 25.7% in 2013 to 35.3% in 2018, although completion rates still remain below target (9). Research has demonstrated protective benefits with even one dose of the vaccine, although the benefit increases with series completion (66). It is imperative that healthcare providers and clinic staff continue to educate patients regarding vaccination schedules and work to identify and minimize barriers to adherence with recommended follow-up. Additionally, to ensure that all patients who are eligible for vaccination have the opportunity to opt in, asking about HPV vaccine status should be included as a standard element of a gynecologic history.

### 4.5 Strengths and limitations

The strength of this study is the diverse patient population across a spectrum of age, race, ethnicity, and nativity. Additionally, the inclusion of Haitian identity allows for the ascertainment of unique disparities in this population. Limitations include survey administration at a single hospital site, limiting generalizability of findings to the national population. Notably, the majority of patients were Hispanic, and a comparatively low number of respondents identified as Haitian. Our surveys were anonymous, and we therefore were unable to link responses with vaccination data, series completion data or cervical cancer screening data in our clinic.

## 5 Conclusions

Our study demonstrates that there are significant deficits in knowledge regarding HPV and HPV vaccination and that HPV vaccination rates remain significantly below national goals in our safety net clinic population.

The lowest percentage of patients reporting knowledge that HPV causes cancer were found among NHO, NHB, HW and Haitian respondents. NHB patients had the highest percentage reporting that they did not know the health consequences of HPV infection. Additional counseling and community-based efforts are needed to address these knowledge gaps. While vaccine uptake did improve following the expansion of access, completion of the vaccination series remains challenging for our patients. Further studies are needed to identify interventions that can improve patient knowledge, vaccine uptake, and provider recommendation for vaccination, and assess barriers to vaccination series completion.

## Data availability statement

The raw data supporting the conclusions of this article will be made available by the authors, without undue reservation.

## Ethics statement

The studies involving humans were approved by Jackson Memorial Hospital Institutional Review Board. The studies were conducted in accordance with the local legislation and institutional requirements. The ethics committee/institutional review board waived the requirement of written informed consent for participation from the participants or the participants’ legal guardians/next of kin because Survey responses were completely anonymous and no chart review was performed. Decision was made by the Jackson Memorial Hospital Institutional Review Board that no written consent was required.

## Author contributions

LF: Conceptualization, Data curation, Formal Analysis, Project administration, Writing – original draft, Writing – review & editing, Methodology. ML: Formal Analysis, Investigation, Writing – review & editing, Data curation, Methodology. AT: Data curation, Writing – review & editing, Formal Analysis, Investigation, Methodology. PJ: Conceptualization, Project administration, Validation, Writing – review & editing, Formal Analysis, Methodology, Resources, Supervision. MH: Conceptualization, Data curation, Formal Analysis, Investigation, Project administration, Resources, Supervision, Writing – review & editing, Methodology.
